# Effect of triage training on nurses with Emergency severity index and Australian triage scale: Α quasi-experimental study

**DOI:** 10.3934/publichealth.2024054

**Published:** 2024-10-09

**Authors:** George Pontisidis, Thalia Bellali, Petros Galanis, Nikolaos Polyzos

**Affiliations:** 1 Department of Social Work, Democritus University of Thrace, University General Hospital of Alexandroupolis, Greece; 2 Department of Nursing, International Hellenic University, Thessaloniki, Greece; 3 Clinical Epidemiology Laboratory, Faculty of Nursing, National and Kapodistrian University of Athens, Athens, Greece; 4 Department of Medicine, Democritus University of Thrace, Alexandroupolis, Greece

**Keywords:** education, triage, nurses, triage education, emergency department

## Abstract

**Introduction:**

Triage training has positive effects on health professionals, the quality of indicators in emergency departments, and the patients. However, data on the effectiveness of triage training on nurses with two different triage scales is limited.

**Objective:**

This study sought to evaluate the effectiveness of a triage training program in Emergency Departments (EDs), as well as the effect on the accuracy, knowledge, and skills of nurses working in the National Health System of Greece.

**Methods:**

Α quasi-experimental study was carried out, with measurements taken pre-, post-, and three months after implementing the education program. Data were collected between March 2021 and July 2022. Eligible participants for this study included nurses employed in the hospital units of the 4^th^ Health Region of the National Health System. A total of 117 nurses participated in the study. Skills, knowledge, and accuracy were assessed using the Emergency Severity Index and the Australian Triage Scale.

**Results:**

After completing the training program, there was a noticeable improvement in the nurses' performance. Their triage skills displayed an overall statistically significant increase (p < 0.001) and, more crucially, in the subscales of rapid patient assessment skills, patient categorization skills, and patient allocation skills. Additionally, statistically significant increases were observed for triage knowledge and for both screening scales that measured triage accuracy, namely the Emergency Severity Index (p < 0.001) and the Australian Triage Scale (p < 0.001). In addition, the number of over-triage and under-triage cases decreased.

**Conclusions:**

The education program had a positive impact on the nurses, resulting in a statistically significant increase in their triage skills and knowledge. Moreover, the use of both triage scales resulted in an increase in the triage accuracy. The increase in triage skills, knowledge, and accuracy decreased after three months.

## 1. Introduction

In recent decades, there has been a growing demand for emergency healthcare services, which has led to overcrowding within emergency departments (EDs) [1]–[4]. As a result, EDs, which are a pivotal part of each hospital, offer health services to patients with both urgent and non-urgent problems [5],[6]. Overcrowding is a multifactorial phenomenon in EDs. The causes have recently focused on three factors: the number of patients coming in (input), the time it takes to process and treat patients (throughput), and the number of patients leaving the ED (output). Other reasons include an increase in the closures of a large number of EDs, the time of year, influenza season, seasonal illnesses, weekends, holiday periods, and COVID-19 [7]. These aforementioned factors can all result in negative consequences. These include an increase in the patient waiting time, patients leaving without being seen (LWBS), mortality, the risk of adverse outcomes, dissatisfaction of the health care staff, treatment costs, and a decrease in the quality of treatment [7].

Health policymakers have been calling upon the government to efficiently address this issue. Triage in the ED is one of the methods that can assist health policymakers in efficiently addressing this problem [1],[2],[8]–[10]

After registering at the secretary's office, patients proceed to the triage area, which is their first interaction with the National Health System (NHS), specifically interacting with healthcare staff [11]. Triage is a dynamic and complex process that ranks patients in the emergency room according to the severity of their illness, not their arrival time [10],[12]–[15].

A triage system has been developed over the last thirty years to help health professionals working in the triage area to make the right decisions and to classify patients according to the severity of their illness. The Australian Triage Scale (ATS), the Manchester Triage System (MTS), the Emergency Severity Index (ESI), and the Canadian Emergency Department Triage and Acuity Scale (CTAS) are five-level decision-making systems that are globally recognized and considered the best [13],[15]–[18].

According to the global bibliography, the majority of healthcare workers who perform triage in EDs have a moderate level of skills and knowledge and do not accurately classify patients according to the available triage system [9],[11],[19]–[27]. Therefore, health professionals who are involved in the triage process should acquire and maintain the appropriate knowledge and skills [11],[28],[29], which can be obtained through education. After basic training, education programs in hospital triage are offered by international organizations such as the Australian Commission for Safety and Quality in Health Care, the Emergency Nurses Association, and hubs. The duration varies depending on the type of program.

A growing body of research conducted in both developed and developing countries shows that triage training has positive effects on health professionals, the quality of EDs, and the patients [6],[30]–[32]. Utilizing repeated measures, Recznik et al. (2019) [33] performed a randomized crossover study with a sample of twenty-five emergency nurses working in the general emergency department in the USA, and found that education had a positive effect on nurses. Nurses more accurately classified the pediatric cases that were the focus of the research. In addition, in a study conducted by McElroy (2020) [32] in a USA hospital ED with a sample of twenty-five emergency nurses and the ESI research tool, it was found that the participants significantly increased the percentages regarding the accuracy of the triage after the education and training in ESI. Furthermore, improvements were also observed in quality indicators of the ED, such as reducing the time needed until the patient was examined and the percentage of patients who left the ED without being seen by physicians.

In Canada, Atack et al. (2006) [34] carried out a study with a sample of 23 nurses that worked in the ED to examine the effect of an online course on the 5-level CTAS on the clinical practice of nurses performing triage. It was found that the online course had a beneficial effect on the nurses' professional development. In a study conducted in a university hospital in Iran by Rahmati et al. (2013) [35] with a sample of 50 health professionals that worked in an ED using the ESI screening scale as a research tool, there was an increase in the health professionals' knowledge after the educational intervention and an improvement in ED quality.

Moreover, a study by Faheim et al. (2019) [8], which was conducted in Egypt with a sample of 150 nurses that worked in the EDs, observed a significant increase in the participants' knowledge and attitudes regarding triage after the educational intervention. In Korea, in studies that contained samples of 27, 25, and 66 nurses respectively [6],[10],[30], it was found that the participants had an improved ability to make clinical decisions, had an increased satisfaction with work and patient orientation [6], had significant improvements in their triage ability and performances, had a reduction in the under-triage percentages [10], and had increased self-efficacy regarding the nurses' classification-categorization of the patients they face acute health problems after the end of the triage education [30]. The aim of this prospective quasi-experimental study is to examine the effectiveness of a triage training program in EDs using the ESI and ATS systems, as well as the effect on the accuracy, knowledge, and skills of nurses working in the Greek NHS.

## 2. Materials and methods

### 2.1. Study design

Research was conducted utilizing a prospective, comparative, and quasi-experimental approach, with only one group included. The data were collected between March 2021 and July 2022. Measurements were taken prior to the education program implementation (1st measurement), immediately following the completion of the training program (2nd measurement), and three months after that (3rd measurement).

### 2.2. Participants

Nurses working in the Greek NHS and, more specifically, in hospital units of the 4th Health Region were eligible to participate. All nurses had been working in hospitals for at least three years. Our nurses were working in a hospital with more than 100 beds. Since all nurses were working in the same Greek health region, they were also working under the same organizational model. The research used convenience sampling to select participants from the readily available population. Specifically, nurses who voluntarily declared their participation in the educational program were included in the study. This method was chosen due to its ease, lack of time required, and low-cost requirements. Nurses who worked in hospital units of the 4th Health Region with either five-month employment contracts or individual employment contracts, employees with Center for Disease Control and Prevention contract status, and those working as auxiliary staff were excluded from the survey. The participants who were employed by hospitals in the 4th Health Region were instructed to fill out the questionnaires for skills, knowledge, and accuracy based on the triage scales employed (ESI and ATS) during all three measurements. The decision to utilize two triage scales instead of one was made to examine the impact of training on two distinct triage scales, which is currently in use worldwide, with the ultimate objective of comparing the screening accuracy between the two scales. The subsequent administration of the questionnaire, which occurred three months after the initial assessment, evaluated the extent to which the knowledge obtained from the educational program had been retained. The individuals enrolled in the training program utilized a unique code number, which was consistently recorded on all three questionnaires, to distinguish and associate the questionnaires with each respective learner. The number of questionnaires indicated refers to the survey participants who completed the questionnaire in its entirety (all three measurement milestones), of which 146 responded at the post-test (second measurements) (146/154; 94.80%) and 117 responded at the 3-month follow-up (117/154; 75.97%).

### 2.3. Intervention program

The duration of the research training program was five academic hours. The structure of the training program was designed based on the Manual for the ESI triage scale [36] and the Triage Education Kit Emergency Triage Workbook for the ATS triage scale [37]. On the one hand, taking family commitments and professional constraints into account, such as nursing shifts and understaffing, the total duration of the program (5 hours) was considered sufficient to ensure a satisfactory participation (longer durations may have hindered participation); on the other hand, the total duration of the program was considered sufficient to ensure that participants received the required training to help them increase their screening skills, knowledge, and accuracy. Based on its design, the theoretical and practical parts of the training program offered the theoretical and practical knowledge necessary to conduct an effective triage. Due to the circumstances of the COVID-19 outbreak, the program was conducted using the e-learning method, often known as e-learning. However, it was originally intended to be implemented in a face-to-face format. The Zoom online platform and modern educational methods were used. Out of the five total hours, the first three hours included the theoretical section, while the final two hours were dedicated to practical applications. The initial three-hour theoretical session of the training program included a detailed presentation of the triage process, including its definition, the historical context, the underlying goals and objectives, the location, the equipment the triage office should have, and a detailed presentation of the most commonly used screening scales worldwide. It analyzed the triage process of adult and pediatric patients (effective and ineffective communication and its consequences), the primary functions and decisions, an evaluation, the outcomes of the triage, the secondary functions and decisions, and the roles and qualifications of the health professionals—triage nurse, triage staff, and legislation. In addition, the five-step and most frequently used screening scales (MTS, ATS, CTAS, and ESI) were presented. Finally, the ATS and ESI triage scales, which were the main research tools, were analyzed in detail. Subsequently, during the final two academic hours, the practical implementation of the screening process was conducted via simulated scenarios, where the participants were actively engaged. The total number of scenarios was forty, of which twenty scenarios involved a triage of patients according to the ATS triage scale and twenty scenarios involved a triage of patients according to the ESI scale. Upon the conclusion of the practical component of the training program, a concise summary of the theoretical portion was provided, followed by a session dedicated to addressing any inquiries.

### 2.4. Variables and instruments

The data were obtained by the administration of a series of questionnaires. The demographic data included information on each individual's characteristics, as well as their educational background and professional experience. Sociodemographic variables and variables related to occupational skills were gathered in the pre-test at the beginning of the study. The variables included gender (male/female), age (continuous variable), work experience (continuous variable), work experience in the ED (continuous variable), and work experience in the triage and training (continuous variable). The triage skills questionnaire (TSQ) is a tool used to assess the participants' screening skills. The TSQ included 37 items that assessed three dimensions: rapid assessment, patient categorization, and patient allocation. The participants were instructed to provide their responses to each item using a scale ranging from 1 to 5. Specifically, a score of 1 indicated a need for improvement, a score of 2 indicated a poor performance, a score of 3 represented an acceptable performance, a score of 4 indicated a good performance, and a score of 5 indicated an excellent performance. The possible range of the total score ranged from 37 to 185. A score below 60% indicates a low level of triage skills, a score between 60–80% indicates a moderate level of triage skills, and a score above 80% indicates a high level of triage skills. The content validity of the questionnaire was evaluated by three experts in Indonesia, and the Cronbach's alpha coefficient was 0.93 [21],[24]. In our study, the Cronbach's alpha coefficient of internal consistency for the TSQ in the first measurement was 0.98, 0.97 in the second measurement, and up to 0.97 in the third measurement, indicating an excellent reliability.

The Triage Knowledge Questionnaire (TKQ) was comprised of a total of 35 multiple-choice questions, each of which had four possible answers. Each successful response was awarded a score of 1, while a wrong response received a 0. The elevated scores are indicative of the nurse possessing a greater level of knowledge. The possible range of the total triage knowledge ranged from 0 to 35. According to the criterion-referenced approach, a score below 60% indicated a poor degree of knowledge, a score between 60% and 80% indicated a moderate level of knowledge, and a score beyond 80% indicated a high level of knowledge. Similar to the TSQ, the content validity of the TKQ questionnaire was evaluated by three experts in Indonesia, and the Cronbach's alpha coefficient was 0.99 [21],[24]. In our study, the Kuder-Richardson internal consistency coefficient for the TKQ in the first measurement was 0.71, 0.77 in the second measurement, and up to 0.74 in the third measurement, indicating an acceptable reliability. Both instruments, TSQ and TKQ, were translated into the Greek language according to the procedure recommended by the “Trust Scientific Advisory Committee” (SAC) [38],[39]. The bilingual translation was performed in two directions (forward-backward translation). Then, we proceeded with the cognitive debriefing process, where we pre-tested the translated questionnaire among a limited group of nurses. To investigate the accuracy according to the triage scale, a case study questionnaire was developed. This questionnaire has two distinct sections (i.e., it includes sixteen case studies), which were evenly distributed between the ESI and ATS classification scales. Each successful response received a score of 1, while an incorrect response received a score of 0. The researcher implemented measures to ensure that the case studies used in the training interventions were evenly distributed. The case studies used in this study were derived from two reputable academic resources: the Agency for Healthcare Research and Quality Handbook for the ESI triage scale [36] and the Australian Government Department of Health and Ageing Emergency Triage Education Kit Triage Workbook for the ATS triage scale [37]. The handbook and workbook are free and easily accessible online. The case studies were translated into the Greek language in the same way as the TSQ and the TKQ.

### 2.5. Data collection

All nurses who participated in the research signed the informed consent form after being informed about their participation in the research prior to the implementation of the training program. Participation was completely voluntary. An initial data collection prior to the implementation of the educational program was conducted, which included variables related to the sociodemographic and occupational variables. After the educational program's implementation, data was collected again at the three-month mark. Data for the three time points was collected using an online questionnaire created in Google Forms and distributed via email. Furthermore, in order to be able to compare the responses in the three phases of the survey and to maintain anonymity throughout the study, the participants were asked to fill in a code in the questionnaire, which would either be the last three numbers of their identity card or their student ID number. In order to maximize the response rate of the participants in the third phase of the survey (i.e., 3 months after the intervention), three reminders were sent to the participants one week apart to encourage them to actively participate in the survey. These reminders contained a hyperlink that provided access to the questionnaire.

### 2.6. Data analysis

The categorical variables are presented as absolute (n) and relative (%) frequencies. In contrast, the continuous variables are presented as the mean, standard deviation, median, minimum, and maximum values. The Kolmogorov-Smirnov test was used to test the normal distribution of the continuous variables. In the analysis of the results, the first measurement corresponds to the measurement before the training, the second measurement corresponds to the measurement after the training, and the third measurement corresponds to the measurement three months after the training. An analysis of variance (ANOVA) for repeated measures was used to investigate the change in the continuous variables across the three consecutive measurements.

Moreover, we performed paired samples t-tests to compare two measurements. Bowker's test was used to examine differences in time regarding nominal variables. The two-sided level of statistical significance was set equal to 0.05. The data analysis was performed using IBM SPSS 21.0 (Statistical Package for Social Sciences).

### 2.7. Ethical considerations

This study adhered to the guidelines outlined in the Declaration of Helsinki [40] and the European Union's standards of good clinical practice. In addition, it first received approval from the scientific boards of the hospitals and then received a final approval from the administrative boards of the hospitals in the fourth health district selected to participate in the training program. The decisions of administrative boards were as follows: University General Hospital of Alexandroupolis: administrative board 12, topic 13/24.03.2021; General Hospital of Kavalas: administrative board 36, topic 30/29–11–2021; General Hospital of Xanthi: administrative board decision 553/22.11.2021; General Hospital of Serres: administrative board 1, topic 8/14.02.2022; General Hospital of Thessalonikis Ippokratio: administrative board 12, topic 16/14.04.2021; General Hospital of Thessalonikis Saint Paulos: administrative board 7, topic 3/16.03.2022; and General Hospital of Komotini: administrative board 44, topic 11/30.09.2021.

## 3. Results

Our sample included 117 nurses. The mean age of the nurses was 41.7 years; most nurses were women (87.2%), and 36.8% of participants had either an MSc or a PhD. The mean years of work experience in health services was 15.9 years, the mean years of work experience in Eds was 5.9 years, and the mean years of work experience in triage offices was 4.5 years. 53% of the participating nurses had experience in an ED. The demographic and professional features of the nurses are presented in Table 1.

### 3.1. Triage skill questionnaire

Descriptive results are presented for the overall skill scores on the three measures in Table 2. The overall mean score of the skills showed a statistically significant increase after education (p < 0.001). Furthermore, the increase is statistically significant from the first to the second measurement (p < 0.001), but not from the first to the third measurement (p = 0.08). From the second to the third measurement, the mean total skill score had a statistically significant decrease (p = 0.001).

The categorization of nurses according to their skill level is presented in Table 3. The percentage of nurses with high triage skills increased, both statistically and significantly, after the education program (p < 0.001).

#### 3.1.1. Triage Skill Questionnaire - Dimension Rapid patient assessment skills

The descriptive statistics results for the rapid patient assessment are presented in Table 2. The mean score for the rapid patient assessment presented skills shows a statistically significant increase after training (p < 0.001). Moreover, the increase is statistically significant from the first to the second measurement (p < 0.001), but not from the first to the third measurement (p = 0.29). The mean skill score regarding the rapid assessment of the patients decreased, both statistically and significantly (p = 0.002), from the second to the third measurement.

**Table 1.** The demographic and professional features of nurses.

Characteristics	Ν	%
Sex
Female	102	87.2
Male	15	12.8
Age^α^	41.7	10.2
Graduated
Technological Education Institute	58	49.6
Higher Educational Institution	16	13.7
MSc/PhD	43	36.8
Entrance to nursing school with
Panhellenic examinations	107	91.5
Qualifying examinations	10	8.5
Working experience in health services	15.9	10.1
Working experience in EDs		
Yes	55	47
No	62	53
Years of working experience in EDs^α^	5.9	6.1
Years of working experience in triage^α^	4.5	3.9
Additional training in the last three years in emergency medicine and nursing
BLS & AED	66	56.4
ILS	18	15.4
ALS	8	6.8
ACLS	3	2.6
BTLS	3	2.6
ATLS	3	2.6
TNCC	0	0
TOC	1	0.9
ECG Resuscitation	0	0
APLS	3	2.6
PLS	4	3.4
Additional training in the last three years
Subsidized training programs	10	8.5
Days Seminar meetings	73	62.4
clinical academic tutorial	50	42.7
Greek congresses	70	59.8
Global congresses	14	12
In-service education courses	44	37.6
Continuing/lifetime education seminars	37	31.6
Speaker in education activities in the last three years	35	29.9
Educator in education activities in the last three years	31	26.5
Member of the organizing/scientific committee in education activities in the last three years	11	9.4
Nursing was the first choice of study		
Yes	53	45.3
No	64	54.7

Note: ^α^Standard Deviation (SD).

#### 3.1.2. Triage Skill Questionnaire - Dimension Patient categorization skills

The descriptive statistics results for the patient categorization skills are shown in Table 2. The mean score for the patient categorization skills shows a statistically significant increase after training (p < 0.001). Additionally, the increase is statistically significant from the first to the second measurement (p < 0.001) and from the first to the third measurement (p < 0.001). From the second to the third measurement, the mean skill score regarding patient categorization decreased, both statistically and significantly (p = 0.004).

#### 3.1.3. Triage Skill Questionnaire - Dimension Patient Allocation skills

The descriptive statistics results for the patient allocation skills are shown in Table 2. The mean score for the patient allocation skills shows a statistically significant increase after training (p < 0.001). Additionally, the increase is statistically significant from the first to the second measurement (p < 0.001) and from the first to the third measurement (p = 0.004). The mean skill score regarding patient allocation decreased, both statistically and significantly (p = 0.001), from the second to the third measurement.

### 3.2. Triage Knowledge Questionnaire

The descriptive statistics results for the triage knowledge are shown in Table 2. The mean score for triage knowledge shows a statistically significant increase after training (p < 0.001). Furthermore, the increase is statistically significant from the first to the second measurement (p < 0.001) and from the first to the third measurement (p < 0.001). The mean knowledge score decreased, both statistically and significantly (p < 0.001), from the second to the third measurement.

The categorization of nurses according to their knowledge level is presented in Table 3. The percentage of nurses with a high triage knowledge increased, both statistically and significantly, after the education program (p < 0.001).

### 3.3. ESI scale

The descriptive statistics results are presented in Table 2 for the ESI scale score on the three measures. The mean accurate triage score shows a statistically significant increase after training (p < 0.001). Moreover, the increase is statistically significant from the first to the second measurement (p < 0.001) and from the first to the third measurement (p < 0.001). The mean accurate triage score decreased, both statistically and significantly (p < 0.001), from the second to the third measurement.

The over-triage with the ESI scale on the three measurements is presented in Table 4. The over triage decreased, both statistically and significantly, from the first to the second measurement (p < 0.001). However, it increased from the second to the third measurement (p < 0.001).

The under-triage with the ESI scale on the three measurements is presented in Table 4. The under-triage decreased, both statistically and significantly, from the first to the second measure (p < 0.001), and it decreased from the second to the third measure, though this was not statistically significant (p = 0.67).

### 3.4. ATS scale

The descriptive statistics results are presented for the ATS scale score on the three measures in Table 2. The mean accurate triage score shows a statistically significant increase after training (p < 0.001). Additionally, the increase is statistically significant from the first to the second measurement (p < 0.001), and from the first to the third measurement (p < 0.001). The mean accurate triage score decreased statistically and significantly (p < 0.001).

The over-triage with the ATS scale on the three measurements is presented in Table 4. The over-triage decreased, both statistically and significantly, from the first to the second measure (p < 0.001), but increased from the second to the third measurement (p < 0.001).

The under-triage with the ATS scale on the three measurements is presented in Table 4. The under triage decreased, both statistically and significantly, from the first to the second measurement (p < 0.001). Furthermore, it increased from the second to the third measurement, but was not statistically significant (p = 0.43).

**Table 2.** Descriptive statistics results for the overall skill scores, rapid patient assessment, and Patient categorization, Patient Allocation, Triage Knowledge, ESI and ATS score on the 3 measures.

	**Οverall triage skill scores**					**p value^α^**
	**mean**	**Standard deviation**	**Median**	**Minimum**	**Maximum**	
First measurement	143.1	27.5	144	55	185	<0.001; First-Second measurement
Second measurement	157.9	19.1	159	94	185	0.08; First-Third measurement
Third measurement	148.9	21.2	150	93	185	0.001; Second-Third measurement
						p<0.001; Multiple comparison
	**Dimension Rapid patient assessment**				
First measurement	105.1	20.7	108	27	135	<0.001; First-Second measurement
Second measurement	114.3	15	114	64	135	0.29; First-Third measurement
Third measurement	107.8	16.2	109	64	135	0.002; Second-Third measurement
						p<0.001; Multiple comparison
	**Dimension Patient categorization**				
First measurement	14.6	3.9	16	4	20	<0.001; First-Second measurement
Second measurement	17	2.3	18	12	20	0.001; First-Third measurement
Third measurement	16.1	2.6	16	10	20	0.004; Second-Third measurement
						p<0.001; Multiple comparison
	**Dimension Patient Allocation**				
First measurement	23.4	5.2	24	6	30	<0.001; First-Second measurement
Second measurement	26.6	3.1	27	16	30	0.004; First-Third measurement
Third measurement	25.1	3.7	26	16	30	0.001; Second-Third measurement
						p<0.001; Multiple comparison
	**Triage Knowledge**				
First measurement	18.8	3.5	19	10	32	<0.001; First-Second measurement
Second measurement	29	2.4	19	22	34	<0.001; First-Third measurement
Third measurement	27.3	2.8	28	19	34	<0.001; Second-Third measurement
						p<0.001; Multiple comparison
	**ESI score**				
First measurement	3.1	1.3	3	1	7	<0.001; First-Second measurement
Second measurement	6.2	0.8	6	4	8	<0.001; First-Third measurement
Third measurement	5.6	0.8	6	4	8	<0.001; Second-Third measurement
						p<0.001; Multiple comparison
	**ATS score**				
First measurement	4.2	1.6	4	1	8	<0.001; First-Second measurement
Second measurement	6.9	0.8	7	5	8	<0.001; First-Third measurement
Third measurement	6.4	0.9	6	5	8	<0.001; Second-Third measurement
						p<0.001; Multiple comparison

Note: ^α^paired samples t-test.

**Table 3.** The categorization of nurses according to their skill and knowledge level.

**Measure**	**Triage skills level**						**p-value^α^**
	**Low (<60%)**		**Moderate (60–80%)**		**High (>80%)**		
	**Ν**	**%**	**Ν**	**%**	**Ν**	**%**	
First	15	12.8	46	39.3	56	47.9	**<0.001**
Second	2	1.7	32	27.4	83	70.9	
Third	6	5.1	44	37.6	67	57.3	
	**Triage knowledge level**						**<0.001**
First	78	66.7	38	32.5	1	0.9	
Second	0	0	29	24.8	88	75.2	
Third	1	0.9	53	45.3	63	53.8	

Note: ^α^Bowker's test.

**Table 4.** Over-triage and Under-triage with ESI and ATS on three measures

	**Over-triage with ESI**					
	**First measure**		**Second measure**		**Third measure**	
	**Ν**	**%**	**Ν**	**%**	**Ν**	**%**
No	52	44.4	84	71.8	30	25.6
Yes	65	55.6	33	28.2	87	74.4
	**Over-triage with ATS**				
No	18	15.4	50	42.7	26	22.2
Yes	99	74.6	67	57.3	91	77.8
	**Under-triage with ESI**				
No	0	0	13	11.1	16	13.7
Yes	117	100	104	89.9	101	86.3
	**Under-triage with ATS**				
No	25	21.4	78	66.7	70	59.8
Yes	92	78.6	39	33.3	47	40.2

## 4. Discussion

Triage, which is a fundamental component of emergency treatment, is a critical nursing function to ensure patient safety and the effective delivery of emergency healthcare services in many countries around the world. In situations where the evidence at hand is insufficient, is comprehensively lacking, or is ambiguous, the triage nurse must cultivate and enhance their critical thinking abilities. Education plays a crucial and essential role in improving the performance of nurses.

The findings of the study showed that the implementation of the education program in triage had a positive effect on the participating nurses. Specifically, there was a statistically significant increase in the triage skills (p < 0.001), knowledge (p < 0.001) and the triage accuracy (p < 0.001) with both the ESI and ATS triage scales between the first and second measurement (immediately after training). In addition, a statistically significant reduction in over- and under-sorting was observed, which significantly contributes to patient safety, since the patient receives the necessary treatment at the right time.

The aforementioned findings exhibit similarities to surveys undertaken in several countries, thus encompassing a representative sample of nurses, physicians, and physician assistants [5]–[8],[10],[12],[29]–[31],[34],[35],[41]–[54].

The findings from these studies demonstrated various statistical significances, except for the study conducted by Campbell et al (2022) [12]. In this study, although there was an observed enhancement in the participants' performance after the training program, the increase in the triage accuracy using the ESI scale, which increased from 78% to 80%, did not have statistical significance.

Comparing the results obtained from the initial and subsequent assessments (i.e., before the start of the educational intervention) and three months after its implementation, it was observed that the participating nurses showed improvements in their screened skills. Therefore, it is vital to highlight that this increase was not statistically significant (p = 0.08) in the overall triage skills and the rapid patients' assessments (p = 0.29) between first and third measurements. Regarding the triage knowledge and accuracy with the ESI and ATS screening scales, the observed increase was statistically significant (p < 0.001) between the first and third measurements. The results of the present study are similar to those of studies conducted in other countries with a sample of nurses, physician assistants, and recently graduated nurses [5],[8],[46],[47],[51],[55]. More specifically, in the study by Ghazali et al. (2020) [5], a statistically significant increase was found between the first and the third measurements (follow-up), in which the Triage Decision Making Inventory (TDMI) questionnaire, p = 0.37, and the Patient Scenario-Based Questions (PSBQs) of adult trauma (AT), p = 0.03, were used. The findings of Bahlibi et al. (2022) [52] align with the aforementioned conclusions. Furthermore, it is worth noting that a statistically significant increase was observed between the initial and the subsequent measurements (follow-up) in terms of the triage knowledge (p < 0.0001), as well as the knowledge of vital signs, waiting time based on urgency classification, and the definition of triage.

The research purpose of Javardi et. al (2023) [46] was to comparatively investigate the effect of different methods, flipped classrooms, and lectures on the nurses' triage knowledge and professional competence. They found a statistically significant increase between the first and the third measurements (follow-up) in screening knowledge (p = 0.001) and the professional competence of nurses (p = 0.001) with both education methods. The more effective method was the flipped classrooms over the lectures.

Toffoli (2016) [51] conducted a study to assess the implementation and effectiveness of an evidence-based, nurse-led, multi-method, cross-disciplinary screening education program to enhance the existing triage procedure. The findings revealed a statistically significant improvement in correct responses between the initial and the follow-up measurements (p < 0.0001). The training program encompassed a combination of online resources, including a 22-minute instructional video on triage in the ESI viewed on YouTube and a four-week training module facilitated through an interactive Facebook group dedicated to emergency department triage. The study encompassed a total of 20 triage scenarios.

The results of a study by Faheim et al. (2019) [8] that investigated the effect of triage teaching on the performance of emergency nurses in various EDs in Egypt led to similar conclusions. The participating nurses' knowledge and practices significantly improved (p < 0.0001) between the first and third measurements (follow-up).

The findings of Mansour et al. (2015) [47] align with those of the aforementioned studies, where they examined the effect of implementing triage training competencies among recently graduated nurses employed in an emergency hospital. The results indicate a noteworthy and statistically significant increase in the screening knowledge (p < 0.001), screening competency (p < 0.001), screening practices, and communication during screening among the cohort of recently graduated nurses, as observed between the initial and the third measurements (follow-up).

Between the second and third measurements, which were conducted three months later in order to investigate whether the acquired skills, knowledge, and screening accuracy remained, the results regarding the participating nurses showed that the acquired skills, knowledge, and triage accuracy did not remain at the satisfactory level of the second measurement, as a decrease was observed. To be more precise, a statistically significant decline was observed in the mean score of the triage skills, knowledge, and correct screening using both the ESI and ATS triage scales between the second and third measurements. There was a significant decline in the total mean score of the triage skills, with the average score dropping from 157.9 to 148.9 (p = 0.001). The mean scores of the dimensions rapid patient assessment, the patient categorization, and the patient allocation significantly reduced from 114.3 to 107.8 (p = 0.002), from 17 to 16.1 (p = 0.004), and from 26.6 to 25.1 (p = 0.001), respectively. Regarding triage knowledge, the mean score decreased from 29 to 27.3 (p < 0.001). The mean score in the triage accuracy with the ESI screening scale decreased from 6.2 to 5.6 (p < 0.001), and the mean score in the screening accuracy with the ATS screening scale decreased from 6.9 to 6.4 (p < 0.001). In addition, an increase in over-triage was observed with both the ESI and ATS screening scales (p < 0.001). In the case of under-triage, an increase was observed with the ATS scale in contrast to the ESI scale, where a further decrease in under-triage was observed, which was not statistically significant (p = 0.67). Considering the results between the second and third measurements, it can be seen that the participating nurses did not maintain the skills, knowledge, and accuracy in triage; however, despite the decrease, the level of remaining knowledge, skills and accuracy in triage remained satisfactory based on the results. To maintain the sorting knowledge, skills, and accuracy to the maximal and desirable degree, they should be updated at regular intervals, either every six months or at least every year, through training programs. Education programs can be developed by the Ministry of Health, NHS hospitals, Universities of Nursing, and nursing organizations. We would like to emphasize the importance of collaboration between these entities in order to ensure comprehensive and effective education for nurses who perform triage in EDs. Similar results were observed in studies conducted in other countries [5],[8],[35],[46],[47],[55]. Compared to the above studies, an increase between the second and third measurements was found in Toffoli (2016) [51] study, as the mean value of correct screening increased from 79.64 to 87.98 (p < 0.0001), which was in direct contrast to our results. The aforementioned findings can be explained by the fact that the participants usually do not have the necessary time to repeat what they learned due to their increased professional and family obligations, especially for the female participants [56],[57]. An additional reason is the lack of support, motivation, and opportunities from both the directorates (medical and nursing) and the hospital administrations [56],[57].

### 4.1. Limitations

The current study is characterized by limitations that may hinder the generalizability of the findings to the entire population of nurses within the country. First, this is a quasi-experimental study. The utilization of convenience sampling as the preferred method for data collection is associated with certain limitations, as this approach inherently results in a voluntary sample. Hence, only individuals who were willing to engage in the research endeavor were included. The current approach did not sufficiently guarantee the representativeness of the study population from which the sample was selected, and it also did not facilitate the generalization of the findings. Furthermore, the absence of a similar or equivalent study within our country poses a barrier in comparing and discussing the results.

## 5. Conclusion

The triage process is of a paramount importance in an ED, as it enhances patient safety and facilitates effective decision-making for resource allocation in urgent and life-threatening cases, thus leading to improved quality of patient care. This education program positively affected the nurses; a statistically significant increase in the triage skills and knowledge was observed. Moreover, there was an increase in the triage accuracy using both triage scales. The increase in the triage skills, knowledge, and accuracy decreased after three months.

Therefore, it is imperative to review the current legislation in Greece with a view to establish an appropriate and efficient triage system, which will substantially contribute to a better use of available resources. The successful deployment of this system relies on training healthcare professionals, especially nurses, since it is necessary to ensure accurate triage and maximize the efficiency.

Nevertheless, evidence is scarce regarding the appropriate training methods for healthcare professionals, particularly in the context of Greece. The current study has endeavored to address this need by demonstrating that using e-learning to train nurses, which encompasses presentations and simulated scenarios, yields a favorable outcome.

While additional research is necessary to examine the potential benefits of this novel strategy to address the issue of overcrowding in EDs and reduce the patients' waiting times, it is essential to note that this study represents the initial investigation into the effectiveness of triage training inside the Greek healthcare system. The study results will be implemented in practical implementation, as well as in the health policy and management of NHS hospitals, in particular to ensure complete and adequate EDs.

Triage systems are a vital part of EDs for patient triage. The National Health System of Greece and other countries can implement similar multi-faceted education programs focused on accurate triage and nurse empowerment.

## Use of AI tools declaration

The authors declare they have not used Artificial Intelligence (AI) tools in the creation of this article.
